# The L-enantiomer of β- aminobutyric acid (L-BAIBA) as a potential marker of bone mineral density, body mass index, while D-BAIBA of physical performance and age

**DOI:** 10.21203/rs.3.rs-2492688/v1

**Published:** 2023-01-23

**Authors:** Charalampos Lyssikatos, Zhiying Wang, Ziyue Liu, Stuart Warden, Marco Brotto, Lynda Bonewald

**Affiliations:** Indiana University; The University of Texas at Arlington; Indiana University; Indiana University; The University of Texas at Arlington; The University of Texas at Arlington

**Keywords:** muscle, physical performance, biomarkers

## Abstract

**Background:**

As both L- and D-BAIBA are increased with exercise, we sought to determine if circulating levels would be associated with physical performance.

**Method:**

Serum levels of L- and D-BAIBA were quantified in 120 individuals (50% female) aged 20–85 years and categorized as either a “low” (LP), “average”(AP) or “high” performer (HP).

Association analysis was performed using Spearman (S) and Pearson (P) rank correlation.

**Results:**

Using the Spearman (S) rank correlation, L-BAIBA positively associated with BMI (0.23) and total fat mass (0.19) in the 120 participants, with total fat mass in the 60 males (0.26) but with both BMI (0.26) and BMD (0.28) in the 60 females. In the HP females, L-BAIBA positively associated with BMD (0.50) and lean mass (0.47).

Using the Pearson (P) rank correlation D-BAIBA was positively associated with age (0.20) in the 120 participants and in the LP females (0.49). D-BAIBA associated with gait speed (S 0.20) in the 120 participants. In HP males, this enantiomer had a negative association with appendicular lean/height (S −0.52) and in the AP males with BMD (S −0.47). No associations were observed in HP or AP females, whereas, in LP females, in addition to a positive association with age, a positive association was observed with grip strength (S 0.45), but a negative with BMD (P −0.52, S −0.63) and chair stands (P −0.47, S −0.51).

**Conclusions:**

L-BAIBA may play a role in BMI and BMD in females, not males, whereas D-BAIBA may be a marker for aging.

## 1. Background

β-aminoisobutyric acid (BAIBA) is an aminobutyric acid (ABA) first discovered in human urine in 1951 (1). It is a small molecule produced by skeletal muscle during exercise (2), and is involved in various metabolic processes such as upregulation of brown adipocyte-specific genes in white adipose tissue, improvement of glucose homeostasis through reducing insulin resistance in skeletal muscle (2), and free fatty acid β-oxidation and energy metabolism by activating the β-oxidation pathway (3). BAIBA has two enantiomers, the L- and D- forms generated by different metabolic pathways (3–5). D-BAIBA is catabolized from thymine and L-BAIBA from the essential branch chain amino-acid valine, which is not endogenously synthesized like thymine. They are also present in different organs; L-BAIBA in the brain, kidney, liver and muscle mitochondria and D-BAIBA in the liver and kidney [3–5].

We have previously shown that L- but not D-BAIBA is produced by murine contracting muscle (6). Our studies in mice have shown that the L-BAIBA is 100 to 1000 times more potent than the D- BAIBA enantiomer in preventing osteocyte apoptosis and that the L form reduces bone and muscle loss due to hindlimb unloading in mice (6). However, in humans, it was reported that the D form was associated with high bone mass in non-osteoporotic females (7). As of now, few studies in humans have investigated the function of BAIBA enantiomers during physical performance.

**Figure 1:**
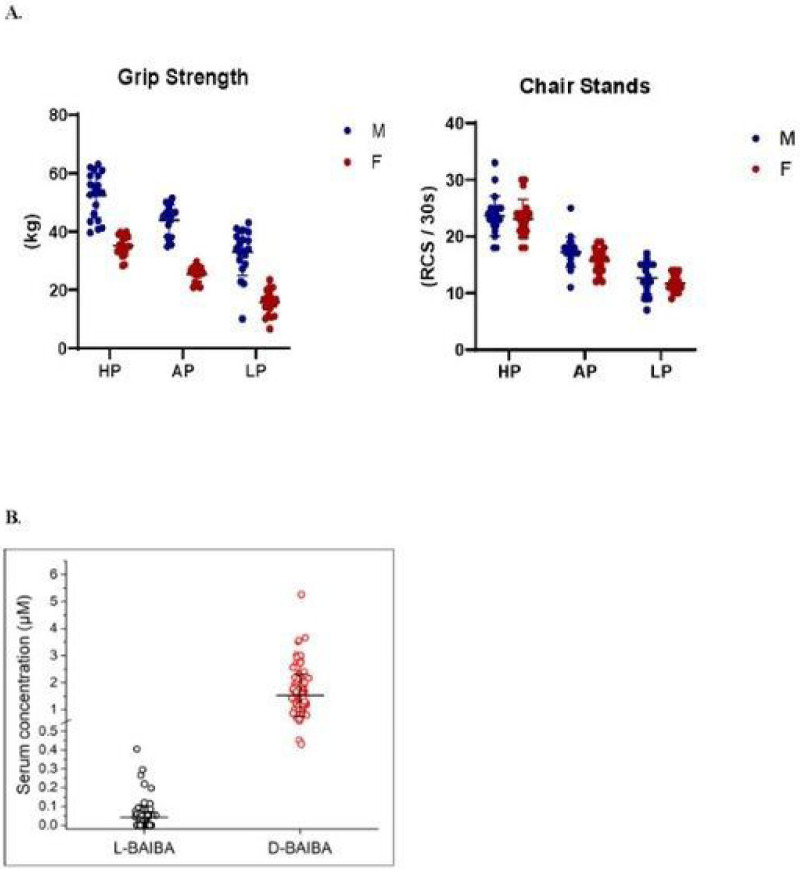
**A.** Classification of performing groups - high [HP], average [AP], low [LP]. Figure legend: Participants were ranked for their performance on best grip strength and number of repeat chair stands completed in 30 seconds. **B.** Comparison of serum *L*-BAIBA levels Figure legend: Comparison of serum *L*-BAIBA levels from the groups containing all participants (n=120) and only with L-BAIBA detectable (n=69). Mean ± SD. *L-BAIBA was detected from 69 out of 120 participants. For the participants with L-BAIBA undetectable in their serum samples, the L-BAIBA levels are counted as “0”.

The aim of this study was to determine if either of the two BAIBA enantiomers was associated with and could therefore serve as a biomarker for physical performance and physical parameters such as BMD and BMI. Serum levels of L- and D-BAIBA were quantitated in 120 individuals aged 20–85 (characteristics table 1), 60 women and 60 men classified as “low”, “average” or “high” performers, (n=20) according to physical performance tests of best grip strength and repeat chair stands (RCS) (Fig. 1A). In the present study, performance was assessed on a set of physical tests as described in methods.

## 2. Methods

### 2.1 Recruitment of human subjects

Serum samples and data were retrieved for 120 individuals who had visited the Musculoskeletal Function, Imaging, and Tissue Resource Core (FIT Core) of the Indiana Center for Musculoskeletal Health’s Clinical Research Center (Indianapolis, Indiana) between 3/2018 and 4/2019. The FIT Core serves to provide: 1) standardized performance of physical function tests and patient reported outcomes related to physical function, 2) imaging outcomes for body composition and bone health, and 3) the collection and banking of biological samples within the Indiana Biobank.

Participants are recruited to the FIT Core by investigators seeking outcomes related to musculoskeletal health for their research subjects, as well as via self-referral from the local community. The Core has Institutional Review Board approval from Indiana University to test all-comers who provide written informed consent, irrespective of current or previous health status.

The FIT Core collected samples and data from 1,518 individuals between 3/2018 and 4/2019. To be included in the current analyses, individuals needed to be 20–85 years of age, self-reported white and non-Hispanic, and without a self-reported major chronic disease. Individuals within each sex and each 15 year age group (20–35, 35–50, 50–65, and 65+yrs) were ranked for their performance on the FIT Core’s hand grip strength test and test of the number of chair stands completed in 30 seconds (Fig. 1A). The 5 individuals within each sex and age range with the lowest, average, and highest composite rank were selected and grouped as low (LP), average (AP), and high (HP) performers, respectively (supplementary tables S1 and S2).

### 2.2 Physical function

The FIT Core assessed dominant hand grip strength (Jamar Plus+digital hand dynamometer; Sammons Preston, Bolingbrook, IL), the number of chair stands completed in 30 seconds, and the time taken to complete 5 chair stands, as we have previously described (8). In addition to raw values, grip strength and repeat chair stand outcomes were converted to age- and sex-matched z scores relative to reference data obtained in the FIT Core (8). Time to walk 4-m from a stationary start at normal speed (usual gait speed) and as quickly as possible without running (fast gait speed) were measured with a stopwatch and converted to speed (m/s), as we have previously reported (9).

Results from the repeat chair stand, usual gait speed, and a static balance test (ability balance for 10 seconds with feet side-by-side, semi-tandem, and tandem) were used to calculate the Short Physical Performance Battery (SPPB) score out of 12 (10). Distance walked in 6 minutes was measured according to the American Thoracic Society (11). The physical function (PF) domain of the NIH Patient Reported Outcomes Measurement Information System (PROMIS) computerized adaptive test (CAT) (PROMIS-CAT-PF) (version 1.2) and the physical functioning subscale of the Short Form 36 (SF-36 PF) were used to assess self-reported functional health.

### 2.3 Body composition and bone health

Appendicular skeletal muscle mass relative to height (ASM/height^2^; kg/m^2^) and whole-body aBMD, fat mass, percent were assessed by whole-body dual-energy x-ray absorptiometry (DXA) (Norland Elite; Norland at Swissray, Fort Atkinson, WI). Regional DXA using the same scanner assessed hip and spine aBMD.

### 2.4 Chemicals and Reagents

Aminobutyric acid standard compounds (S)-3-aminoisobutyric acid (*L*-BAIBA) and (R)-3-aminoisobutyric acid (*D*-BAIBA) were purchased from Adipogen Corp. (San Diego, CA). G. Isotopic internal standard (IS) compounds (±)-3-amino-iso-butyric-2,3,3-d_3_ acid (*D*,*L*-BAIBA-d_3_) were obtained from CDN Isotopes (Pointe-Claire, Quebec, Canada). Formic acid (reagent grade, ≥ 95%), Bovine Serum Albumin (BSA) were obtained from Sigma–Aldrich (St. Louis, MO). Phosphate Buffered Saline (PBS) was purchased from Fisher Scientific (Pittsburgh, PA). HPLC-MS grade acetonitrile, water, and methanol were purchased from J.T. Baker (Phillipsburg, NJ).

### 2.5 LC-MS/MS conditions

All components of liquid chromatography-tandem mass spectrometry (LC-MS/MS) system are from Shimadzu Scientific Instruments, Inc. (Columbia, MD). The LC system was equipped with pumps A and B (LC-30AD), and autosampler (SIL-30AC). The LC separation was conducted on a chiral SPP-TeicoShell column (150 × 4.6 mm, 2.7 μm, AZYP LLC., Arlington, TX) configured with a Synergi^™^ 4 μm Max-RP column as guard column (50 × 2.0 mm, Phenomenex, Torrance, CA). The MS/MS analysis was performed on Shimadzu LCMS-8050 triple quadrupole mass spectrometer.

Quantification of isomeric aminobutyric acids in human serum samples was followed the LC-MS/MS method (7). Brie y, mobile phases are methanol (A) and water containing 0.005% formic acid and 2.5 mM ammonium formate (B). The MS instrument was operated and optimized under positive electrospray (+ESI) and multiple reaction monitoring modes (MRM). The *m/z* transitions (precursor to product ions) and their tuning voltages were selected from published paper (7) and further optimized based on the best MRM responses from instrumental method optimization software. All analyses and data processing were completed on Shimadzu LabSolutions V5.91 software (Shimadzu Scientific Instruments, Inc., Columbia, MD).

### 2.6 Sample Preparation for LC-MS/MS analysis

Ten microliter human serum samples and same volume of IS mixture solution (1.2 μM, 0.1% formic acid in methanol, v/v) were added to 35 μL 0.1% (v/v) formic acid in methanol, followed by 20 min-shaking at room temperature and another 15 min-centrifugation at 15,000 ×g, 4°C to precipitate the proteins. The supernatant was directly transferred to autosampler vial and 45 μL of each sample was injected for LC-MS/MS analysis.

The samples of standard calibration curves were prepared by spiking the pure standards in surrogate matrix 5% (w/v) BSA in PBS (pH7.4). The samples for ten-point calibration curves were prepared by diluting the working solution to 0.02–10.24 μM for L-BAIBA and D-BAIBA. Then ten microliters of each standard sample were taken and treated following the same preparation procedures of serum samples for LC-MS/MS analysis.

### 2.7 Statistical analysis

Data were summarized as mean±SD. Comparisons among groups were performed using Student’s t-test and one-way ANOVA with Tukey’s post-Hoc test (α=0.05). Association analysis was performed using both Pearson (P) correlations and Spearman (S) correlations. To control for the effects of age and BMI, partial correlations were further calculated. SAS 9.4 (SAS Institute, Cary, NC, USA) was used for statistical analysis. Two-sided p-values<0.05 were considered as significant. Both Pearson (P) and Spearman (S) correlations were examined for associations with D-BAIBA to characterize these associations more extensively than a single correlation type, while Spearman correlation is more robust to outliers and able to capture nonlinear but monotonic associations (Pearson correlation captures the linear ones better). For this reason, for L-BAIBA statistical analysis only the Spearman correlations were used. For those with L-BAIBA undetectable in their serum samples (some L-BAIBA levels were under the limit of detection and their values could not be determined) the L-BAIBA levels are counted as “0”. Heatmaps were generated to display correlations with the magnitude coded by colors and numbers been displayed for the ones with p value<0.05.

## 3. Results

### 3.1 Participant characteristics

There were no age group X function group interactions for any participant characteristics (all *p*=0.06 to 0.34). Grip strength and chair stands completed in 30-s in both females and males were lower with higher age group and lower function group (all *p* ≤ 0.001). The average hand grip z-score in LP, AP, and HP across both sexes was −1.22 (95% confidence [CI], −1.42 to −1.02), 0.10 (95% CI, −0.10 to 0.30), and 1.36 (95% CI, 1.17 to 1.56), respectively. The average z-score for chair stands completed in 30-s in LP, AP, and HP across both sexes was −1.15 (95% confidence [CI], −1.32 to −0.98), 0.07 (95% CI, −0.10 to 0.25), and 1.53 (95% CI, 1.36 to 1.70), respectively.

The concentrations for D-BAIBA and L-BAIBA in the study population ranged from 1.53 +/− 0.77 μM and 0.043 +/− 0.0060 μM, respectively (Fig. 1B).

L-BAIBA concentrations with the different physical performing populations (LP: low performers, AP: average performers, HP performers) were not statistically significant and are summarized in Table 2a. Similarly, no statistically significant difference was noticed between the performers’ groups with the four age groups (20–35, 35–50, 50–65 and older adults 65+) (Table 2b) and gender (Table 2c).

### 3.2 Correlations of physical performance and physical parameters in all 120 samples, 60 females and 60 males, age 20–85

**Figure 2:**
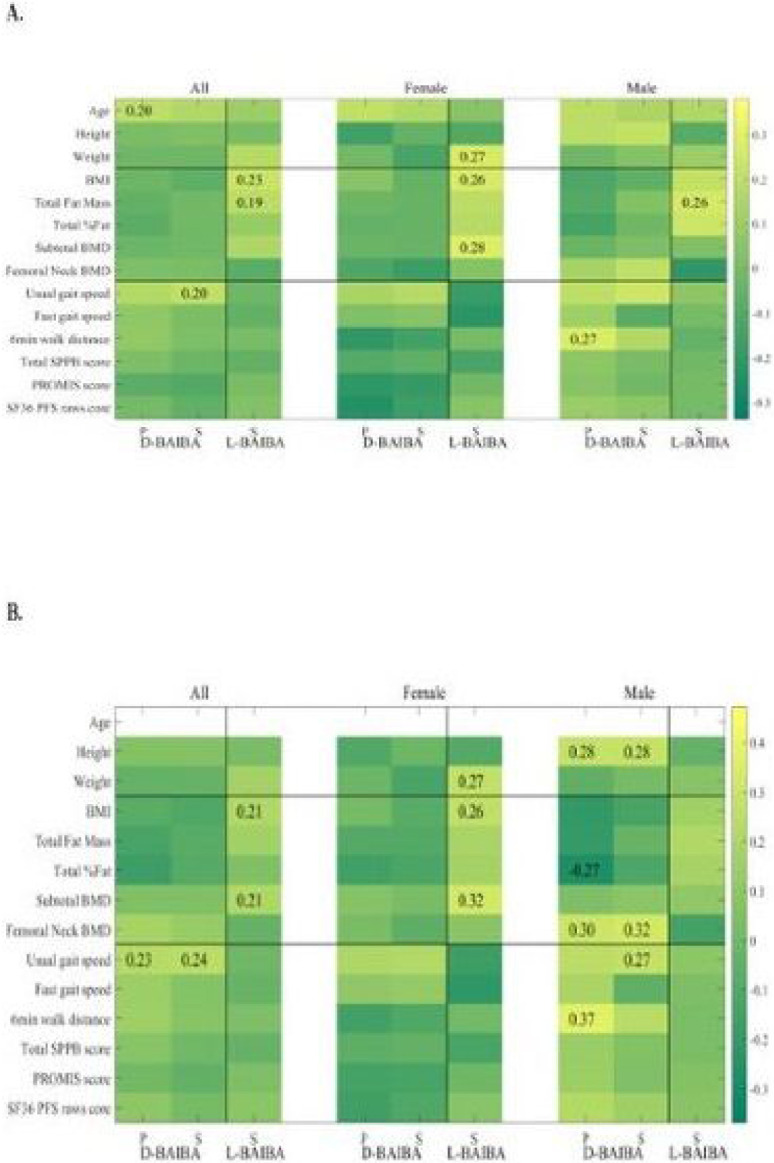
**A.** Heatmap of D- and L- BAIBA with age and characteristics of physical performance Figure legend: in all 120 samples, 60 females and 60 males, age 20–85. **B.** after the age effect was removed (P- Pearson and S- Spearman).

When all 120 samples (60 females and 60 males, age 20–85) were examined, D-BAIBA had a positive Pearson (P) correlation with age (0.20, p<0.05) and positive Spearman (S) association with usual gait speed (0.20, p<0.05) (Fig. 2A and **supplementary table S3a**). L-BAIBA had a positive Spearman association with BMI (0.23, p<0.05) and with total fat mass (0.19, p<0.05) (Fig. 2A and **supplementary table S3b**). No significant correlations were observed with any other parameters.

When the 120 participants were divided between females and males, D-BAIBA in males had a positive Pearson correlation (0.27, p<0.05) with the 6MWT (Fig. 2A).

In the 60 female participants, L-BAIBA had a positive Spearman association with BMI (0.26, p<0.05), weight (0.27, p<0.05) and subtotal BMD (0.28, p<0.05) (Fig. 2A and **supplementary table S4a**). L-BAIBA in the 60 males was positively related with total fat mass (0.26, p<0.05) (Fig. 2A and **supplementary table 4b**). No significant correlations were observed with any other parameters.

With the age effect removed most of the above correlations remained significant; and D-BAIBA in males had a positive P (0.30) and S (0.32) correlation with femoral neck BMD and positive S (0.27) with usual gait speed (Fig. 2B and **supplementary table S5**). A positive D-BAIBA association with femoral neck BMD in males was seen after the age and BMI effects (P 0.32, p<0.05, S 0.33, p<0.05) were removed (**supplementary Fig. 1A** and **supplementary tables S7 and S8**).

### 3.3 Correlations of physical performance and physical parameters in HP, AP and LP

**Figure 3:**
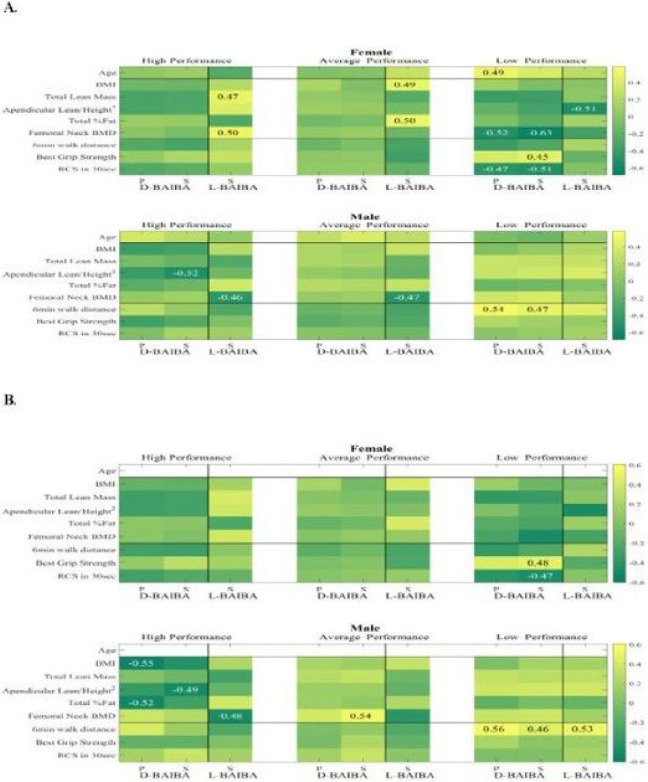
**A.** Heatmap of D- and L- BAIBA with age and characteristics of physical performance Figure legend: in HP, AP and LP in Females and Males. **B.** after the age effect was removed (P- Pearson and S- Spearman).

High performing (HP) females’ L-BAIBA had a positive Spearman correlation with femoral neck BMD (S 0.50, p<0.05) and total lean mass (0.47, p<0.05) (Fig. 3A). This positive correlation with femoral neck BMD was opposite from what was found in HP males; and remained significant after age and BMI effects had been removed (0.48, p<0.05, **supplementary Fig. 1B**). Also, there was a negative L-BAIBA association with the total fat percent, after age and BMI effects were removed (−0.53, p<0.05) (**supplementary Fig. 1B** and **supplementary table S4a**). No significant correlations were observed with any other parameters.

In the group of HP males, D-BAIBA had a negative association (S −0.52, p<0.05) with the appendicular lean/height^2 (kg/m^2) (Fig. 3A and **supplementary table S5**). L-BAIBA had a negative relation with the femoral neck BMD (S −0.46, p<0.05). Both these associations remained significant after age effect had been removed (−0.49 and −0.48, respectively) (Fig. 3B).

In **average performing** (AP) females, L-BAIBA had a positive association with BMI (S 0.49, p<0.05) and total fat percent (0.50, p<0.05) (Fig. 3A and **supplementary table S4a**), and in males, L-BAIBA had a negative association with the femoral neck BMD (−0.47, p<0.05) (Fig. 3A and **supplementary table S4b**).

In the **low performing** (LP) females, D-BAIBA had a positive correlation with age (P 0.49, p<0.05), positive correlation with best grip strength (S 0.45, p<0.05), negative associations by both Pearson and Spearman with the RCS in 30 sec (P-0.47, p<0.05 and S −0.51, p<0.05), and femoral neck BMD (P – 0.52, p<0.05, S – 0.63, p<0.05) (Fig. 3A). The Spearman correlations for best grip strength (ρ 0.48, p<0.05) and RCS in 30 sec (ρ −0.47, p<0.05) remained significant after age effect had been removed (Fig. 3B and **supplementary table S6**). Detailed D-BAIBA correlations in LP females with physical performance parameters are shown in the **supplementary tables S9** and **S10**.

Regarding the L-BAIBA in the LP females, there was a negative association with appendicular lean/height^2 (kg/m^2) (−0.51, p<0.05) (Fig. 3A), that remained significant after age and BMI effects had been removed (−0.52, p<0.05) (**supplementary Fig. 1B** and **supplementary table S4a**).

D-BAIBA in LP males had a positive correlation with 6MWT (P 0.54, p<0.05 and S 0.47, p<0.05) (Fig. 3A). This correlation remained significant after the age effect had been removed (P 0.56, p<0.05, and S 0.46, p<0.05) (Fig. 3B, **supplementary tables S7** and **S8**).

L-BAIBA in the LP males’ group, had a positive association with the 6MWT (S 0.53, p<0.03) after age effect had been removed (Fig. 3B and **supplementary table S4b**). No significant correlations were observed with any other parameters.

## 4. Discussion

Muscle and bone communicate through the production of myokines and osteokines (12). Contracted muscle and loaded bone produce beneficial signaling molecules while unloaded muscle and resorbing bone produces molecules that have negative effects on the opposing tissue. Myokines can have autocrine, paracrine, and endocrine functions (13). An example of a negative myokine is myostatin also known as growth and differentiation factor 8 (GFD-8), a ubiquitous glycoprotein that negatively regulates skeletal and cardiac muscle, and reduces bone mass by stimulating osteoclast activity (14) through the activin type IIB receptor (ActRIIB) (15). An example of a positive myokine is irisin, released by the cleavage of the extracellular domain of the transmembrane receptor bronectin II domain-containing 5 (FNDC5) (16), and has been found to be associated with browning of white adipose tissue and with energy metabolism (13). Several other factors are produced by sedentary and contracted muscle not reviewed here. {For review see (12, 17)}.

Conversely, bone produces osteokines such as osteocalcin, encoded by the *BGLAP* gene with a unique G-protein coupled receptor -GPRC6A that has a significant role in energy metabolism (glucose and fatty acids), in adipose, muscle and neuronal tissue (12, 18). Osteocalcin together with IL-6 from skeletal muscle increase adaption during exercise by providing the necessary carbon atoms to the TCA cycle (19). PGE_2_ is an eicosinoid made by loaded osteocytes that stimulates proliferation of satellite cells (muscle stem cells) through upregulation of the peroxisome proliferator-activated receptor-γ coactivator 1α (PGC-1 α) expression (20). Osteocytes are a major source of PGE_2_ especially in response to mechanical loading and secrete 1,000–10,000 times the amount produced by muscle cells (12).

β-aminobutyric acid (BAIBA) is a small non-protein metabolite. In humans, BAIBA has a metabolic effect by reducing insulin resistance (2). Several studies in animal models and humans have measured total BAIBA, but not its enantiomers. We showed that murine contracted muscle produces the L but not the D-enantiomer and that L-BAIBA has a protective effect on bone (6). A recent study of heart failure in rats showed that L-BAIBA produced during treadmill exercise has a protective effect on cardiac muscle cells (21).

The synergistic action of bone and muscle during exercise allows standing and moving – physical performance - and leads to movement and energy consumption (22). Exercise represents the planned, structured and repetitive physical activity with the goal to improve physical fitness. It is divided in two types: aerobic or endurance and anaerobic or resistance (22). It is not known which form or combination is most conducive to the production of BAIBA and if any associations exist for bone and muscle health.

A study in Native American boys and girls (11–17 years) divided into obese and normal-weight groups, measured blood BAIBA levels after 16 weeks of aerobic exercise training and showed that the normal-weight group had 29% higher BAIBA levels than those in the obese group (23). Physical inactivity in patients on hemodialysis has been found to reduce plasma BAIBA concentrations (24, 25), and plasma BAIBA levels are higher in young subjects than the elderly (26). This could be because BAIBA expression is regulated by PGC-1α, and PGC-1α expression is lower in elderly subjects compared to young subjects (27, 28). Additionally, high plasma BAIBA concentrations were observed in humans undergoing aerobic exercise but are inversely correlated with metabolic risk factors, suggesting that BAIBA may protect against metabolic diseases (2, 29–31).

For this retrospective single center study, our hypothesis was that physical performance would increase the BAIBA enantiomers levels and therefore serve as a biomarker for high performance. The primary aim was to determine if a correlation existed between L- or D- BAIBA with different physical tests in both males and females from different ages (20–34, 35–49, 50–64, 65+) and different levels of physical performance (LP, AP and HP groups). A study by Roberts et al performed with 80 healthy participants, both genders, mean age of 34 years, previously sedentary, with a 20-week aerobic training, showed a total BAIBA increase of 17% (2). A 2016 study in 49 patients with chronic kidney disease on hemodialysis, showed that BAIBA (probably L-BAIBA, based on what was mentioned in the manuscript that the BAIBA is generated from valine) was lower in hemodialysis physically inactive patients compared to active patients (able to exercise at least once a week) (24). The first clinical study (pubmed search till June of 2022) where the enantiomers and not total BAIBA were quantitated was in 2019 and showed that L-BAIBA increased during aerobic exercise and continued to increase at 30 min after participants completed the exercise schedule. D-BAIBA also increased during exercise but dropped at 30 min after the exercise schedule (30). In 2017 a cyclic exercise study with 13 untrained male participants did not show an increase of total BAIBA during exercise, however, a small increase was noted at 4 hours (32).

In the present study, for the 120 total participants, L-BAIBA positively correlated with BMI and total fat mass. But BMI is a rough estimate of relative body weight, not accurate for body composition and does not include fat mass (33). In the 60 male participants, L-BAIBA associated with total fat mass and in the 60 female participants, with BMI and subtotal and total BMD. With regards to physical performance, in the male high performing group, L-BAIBA was negatively linked with femoral neck BMD whereas in the female high performing group, L-BAIBA had a positive association with femoral neck BMD and lean mass. In the average male performers, L-BAIBA had a negative association with femoral neck BMD, while in females, L-BAIBA was positive with BMI and total % fat. L-BAIBA had a positive association with 6MWT in low performing males, but a negative with appendicular lean/height^2 (kg/m^2) in low performing females. It was surprising that L-BAIBA would be so strongly associated with parameters of fat mass. However, with high physical performance in females, this was reversed and became associated for BMD and lean mass. This suggests another function of L-BAIBA distinct from mediating the effects of exercise.

For the 120 total participants, D-BAIBA positively associated with age and gait speed. In the 60 male participants, D-BAIBA positively associated with femoral neck BMD and six-minute walk test (6MWT). With regards to physical performance, in the male high performing group, D-BAIBA had a negative correlation with appendicular lean/height^2 (kg/m^2). In the low performing males, D-BAIBA had a positive correlation with 6MWT. It is not clear why this enantiomer would be associated with age. Also, it is not clear why D-BAIBA would be positively associated with the 6MWT. Additional studies will be required.

Data analysis of the 120 de-identified human serum samples in this cohort, did not support the hypothesis of elevated L-BAIBA in high performing individuals. Although the chosen samples for analysis were from study participants without a chronic disease – not on prescription medications (for instance not on statins which are known to be associated with myopathy); there are limitations. The retrospective nature of the study from already collected blood samples, a single center study and the relatively small cohort of 120 samples may account for the lack of significance. A major limitation may be not knowing how soon after exercise the blood was drawn. It has recently been described that BAIBA secretion increases during and after exercise (34). Other confounding factors that could be considered include circadian rhythm, diurnal variation/time of the day in which blood samples were collected, the fact that the blood draws were either at the beginning or the end of the physical performance tests, exercise status is unknown, as is the use of vitamins and /or supplements. Hormone replacement or use of contraception was unknown in the samples collected from female participants. Diet was not controlled before participating in the study and dietary history was not collected. Also, the conversion of D-BAIBA to L-BAIBA and vice versa during exercise cannot be excluded. Clinical studies with measurement of BAIBA and its enantiomers during resistance exercise have not been performed to date. A future study where most of the above factors can be controlled, such as a study in healthy young untrained adults where the enantiomers and other ABAs and metabolites can be measured during and after the two different types of exercise (aerobic and resistance) could provide more information regarding the release, timing, and response to exercise.

## 5. Conclusions

In summary, L-BAIBA did not show major associations with physical performance except for 6MWT but did show significant associations with the physical characteristics of BMI, fat mass, lean mass, and femoral neck BMD, while D-BAIBA showed significant associations with age and physical performance (usual gait speed, 6MWT, grip strength, RCS) features. The mechanisms responsible for these associations remain to be explored.

## Figures and Tables

**Table 1.** Characteristics of the 120 participants

	N	Mean	SD	Median	Min	Max
Age (years)	120	49.60	17.58	50.08	20.09	84.73
Height (cm)	120	170.70	8.91	169.55	149.00	191.80
Weight (kg)	120	78.13	13.62	77.80	54.10	114.10
BMI (kg/m^2^)	120	26.83	4.48	25.55	19.70	45.10
Appendicular lean/height^2^ (kg/m^2^)	117	7.84	1.39	7.98	4.64	12.14
Total BMD (g/cm^2^)	116	1.11	0.15	1.11	0.77	1.43
Spine BMD (g/cm^2^)	117	1.09	0.17	1.09	0.68	1.45
Femoral Neck BMD (g/cm^2^)	117	0.89	0.17	0.85	0.55	1.35
Total SPPB score	120	11.56	1.38	12.00	0.00	12.00
SPPB gait speed score	120	3.99	0.09	4.00	3.00	4.00
Usual gait speed (m/s)	120	1.40	0.18	1.40	0.74	2.00
Fast gait speed (m/s)	120	2.05	0.34	2.05	1.01	3.28
Best Grip Strength (kg)	120	34.53	12.80	34.85	6.60	63.00
Time for 5 chair stands (s)	120	8.87	2.96	8.08	2.13	19.77
6-min walk distance (m)	117	569.62	109.81	580.00	160.00	969.00
PROMIS score	120	56.42	7.27	54.70	40.40	73.30
SF-36 PFS raw score	120	92.96	12.79	100.00	20.00	100.00

**Table 2a.** Serum concentrations of L- and D-BAIBA in different physical performance populations

Aminobutyric acids	HP	AP	LP	Overall
Sample size N	40	40	40	120
*L*-BAIBA (μM)	0.034 ± 0.030	0.054 ± 0.096	0.040 ± 0.030	0.043 ± 0.060
*D*-BAIBA (μM)	1.65 ± 0.94	1.46 ± 0.61	1.49 ± 0.72	1.53 ± 0.77

**2b.** Serum concentrations of L- and D-BAIBA in different age populations

Aminobutyric acids	Measurements	Physical performance	Age group				
			20–34 yrs	35–49 yrs	50–64 yrs	65+ yrs	p-value
*D*-BAIBA (μM)	All participants in each group	All	1.38 ± 0.62 (30)	1.51 ± 0.65 (30)	1.47 ± 0.67 (30)	1.77 ± 1.03 (30)	0.2374
		High (HP)	1.54 ± 0.79 (10)	1.43 ± 0.40 (10)	1.56 ± 0.87 (10)	2.07 ± 1.41 (10)	0.4432
		Average (AP)	1.31 ± 0.56 (10)	1.63 ± 0.69 (10)	1.38 ± 0.66 (10)	1.51 ± 0.56 (10)	0.662
		Low (LP)	1.30 ± 0.53 (10)	1.48 ± 0.83 (10)	1.46 ± 0.48 (10)	1.73 ± 0.98 (10)	0.6314
*L*-BAIBA (μM)	All participants in each group^1^	All	0.025 ± 0.028 (30)	0.041 ± 0.044 (30)	0.052 ± 0.066 (30)	0.052 ± 0.085 (30)	0.2572
		High (HP)	0.037 ± 0.030 (10)	0.038 ± 0.036 (10)	0.029 ± 0.028 (10)	0.033 ± 0.030 (10)	0.9048
		Average (AP)	0.0052 ± 0.016 (10)	0.047 ± 0.064 (10)	0.081 ± 0.106 (10)	0.084 ± 0.140 (10)	0.2243
		Low (LP)	0.033 ± 0.027 (10)	0.038 ± 0.028 (10)	0.048 ± 0.027 (10)	0.039 ± 0.038 (10)	0.7553

For the participants with L-BAIBA undetectable in their serum samples, the L-BAIBA levels are counted as “0”.

**2c.** Serum concentrations of L- and D-BAIBA in different gender populations

Aminobutyric acids	Measurements	Physical performance	Gender		
			Female	Male	p-value
*D*-BAIBA (μM)	All participants in each group	All	1.55 ± 0.76 (60)	1.52 ± 0.78 (60)	0.8306
		High (HP)	1.58 ± 0.70 (20)	1.72 ± 1.14 (20)	0.6427
		Average (AP)	1.51 ± 0.71 (20)	1.40 ± 0.51 (20)	0.5449
		Low (LP)	1.55 ± 0.88 (20)	1.44 ± 0.53 (20)	0.6291
*L*-BAIBA (μM)	All participants in each group^1^	All	0.037 ± 0.064 (60)	0.048 ± 0.064 (60)	0.3348
		High (HP)	0.036 ± 0.031 (20)	0.033 ± 0.030 (20)	0.8138
		Average (AP)	0.036 ± 0.092 (20)	0.072 ± 0.098 (20)	0.2421
		Low (LP)	0.040 ± 0.024 (20)	0.039 ± 0.035 (20)	0.8848

Each data represents mean ± SD (n). ANOVA was applied for statistics analysis. *p<0.05.

For the participants with *L*-BAIBA undetectable in their serum samples, the *L*-BAIBA levels are counted as “0”.

## Data Availability

All data generated and analyzed during this study are included in this published article and its supplementary information files.

## Abbreviations

6MWT: six-minute walk test
ABAs: aminobutyric acids
BAIBA: β-aminoisobutyric acid
BMD: bone mineral density
BMI: body mass index
BSA: bovine serum albumin
ESI: electrospray Ionization
IS: Internal standard
LC: liquid chromatography
LC-MS/MS: liquid chromatography-tandem mass spectrometry
MRM: multiple reaction monitoring
MS: mass spectrometry
m/z: mass-to-charge ratio
PBS: phosphate-buffered saline
MSK: musculoskeletal
SD: standard deviation
